# Digital Storytelling Intervention for Enhancing the Social Participation of People With Mild Cognitive Impairment: Co-Design and Usability Study

**DOI:** 10.2196/54138

**Published:** 2024-01-17

**Authors:** Di Zhu, Abdullah Al Mahmud, Wei Liu

**Affiliations:** 1 Centre for Design Innovation Swinburne University of Technology Melbourne Australia; 2 Beijing Key Laboratory of Applied Experimental Psychology National Demonstration Centre for Experimental Psychology Education, Beijing Normal University Faculty of Psychology, Beijing Normal University Beijing China

**Keywords:** co-design, digital storytelling, people with mild cognitive impairment, MCI, technology-based intervention development, dementia, mobile phone

## Abstract

**Background:**

Community-based social participation has shown promise in delaying cognitive decline in older adults with mild cognitive impairment (MCI) who are at risk of developing dementia. Although group storytelling interventions have proven effective, the need for a skilled workforce to support people with MCI can limit broader community implementation. Technology-based interventions may offer a solution to this limitation by replicating the abilities of therapists.

**Objective:**

This study aims to co-design a digital storytelling intervention and evaluate its usability.

**Methods:**

This co-design process involved 3 stages, engaging people with MCI (n=12), their caregivers (n=4), and therapists (n=5) in Beijing, China. In the first stage, we used card sorting and voting methods to identify potential incentives for social participation and target the specific abilities that people with MCI wanted to enhance. In the second stage, we conducted brainstorming sessions with people with MCI and their caregivers to identify the potential features of a digital storytelling application named Huiyou (“meeting new friends” in Chinese). Finally, we assessed Huiyou’s usability with people with MCI and therapists, leading to iterative improvements based on the usability findings.

**Results:**

We uncovered a crucial link between boosting the self-confidence of people with MCI and their ability to address social participation challenges. Notably, we identified memory improvement and enhanced language expression as key factors for effective communication with grandchildren. Subsequently, participants suggested features and interfaces to address these challenges, leading to the development of Huiyou, a group-based digital storytelling application featuring functions such as generating story materials, conducting memory retrieval activities, and sharing stories. It received an “excellent” rating in the User Experience Questionnaire benchmark, displaying high levels of attractiveness, dependability, stimulation, and novelty. People with MCI achieved an average task completion rate of 87% (n=19; SD 0.13) of the 22 tasks. However, feedback from people with MCI and therapists highlighted usability issues in navigation, activity management, user interface, and feature optimization, indicating a need for improved accessibility and efficiency.

**Conclusions:**

The co-design approach contributed to developing the Huiyou prototype, supporting community-based social participation. User feedback highlighted the potential of Huiyou to enhance well-being and facilitate meaningful social interactions while maintaining crucial existing relationships.

## Introduction

### Background

Social participation has proven efficacious in decelerating the progression of dementia from mild cognitive impairment (MCI) [1], particularly in low- and middle-income countries [2]. To enhance social participation among older adults with MCI, researchers are engaged in reducing the obstructions to social participation [3] and creating and customizing a variety of constructive social activities [4]. To expand social participation, researchers have explored an assortment of interventions aimed at improving memory, communication, and familial relationships among older adults with MCI [5]. Social participation is considered at different levels: societal, community, relationship, and individual levels [6]. Community involvement is defined by its significance and the sense of satisfaction it generates in people’s lives as well as its potential for creating social support [7]. Older adults reported that technology could assist them in maintaining social connections [8]. For example, researchers used cameras, enabling people with MCI to memorialize their daily lives [9]. Most interventions concentrate on enhancing cognitive abilities [10] or facilitating recreational and sports activities [11]. The former relies upon participants’ strong motivation to participate in interventions, whereas the latter has less impact on cognitive ability. Storytelling tools can simultaneously enhance cognitive abilities and create a novel form of social interaction. Incorporating storytelling as a therapeutic intervention possesses enormous potential for improving health care outcomes and promoting well-being among people with MCI [12]. Studies have demonstrated that socially isolated people with MCI may experience lower psychological well-being and exhibit more negative states of mind than older adults considered to have cognitive normality [13]. Owing to cognitive impairments, people with MCI may encounter the following issues: (1) reduced interest in and initiation of social interactions, suggesting a lack of proactive engagement [14]; (2) difficulties in establishing and maintaining interpersonal relationships, implying an impaired sense of social synchrony; and (3) challenges in recognizing and adhering to social boundaries and norms [15]. It is imperative to explore behavior change strategies aimed at fostering a positive social health lifestyle among people with MCI. Technology could be a beneficial tool to maintain social connection [8]. For example, ElderConnect is a web-based app designed to assist senior citizens in recognizing, preventing, and easing feelings of loneliness. It offers information and tactics to help them establish new social connections and sustain existing ones [16]. Notably, storytelling serves as an essential activity for promoting positive social health [17,18]. Harnessing the power of storytelling, people with MCI can be encouraged to participate more proactively in their health care by identifying their unique needs and knowledge gaps while fostering strong connections and support networks with peers with similar disease-related experiences [19].

However, these storytelling interventions often lack a focus on community-based social activities, primarily being individual interventions without emphasis on interpersonal interactions. Furthermore, community-based programs depend on skilled facilitators, such as therapists or social workers, to maintain quality. For instance, providing training for facilitators before they lead a group reminiscence program ensures expertise in techniques and effective program management [20]. In integrative group storytelling therapy, participants exhibited strong social integration, supported one another, displayed valuable life skills, and fostered positive self-worth and institutional loyalty during reminiscing and feedback sharing [21]. Consequently, participants felt supported and experienced a sense of belonging when performing digital group activities [22]. Community-based social participation relies on facilitators who organize activities and provide support in securing spaces and promoting events. Therefore, using a technology-based storytelling intervention to boost community-based social engagement can enhance community belonging.

The 3 main features of a digital storytelling application are multimedia material editing, memory recall, and story sharing. Some memory retrieval interventions may combine multiple functions of the interventions, such as people with MCI simultaneously reminiscing and sharing their recollections [23]. Challenges in using technology remain an unsolved issue; people with MCI typically exhibit lower digital literacy [24]. Most storytelling programs include training sessions before the intervention, such as storytelling classes [25], as mostly commercial, off-the-shelf technology is used to support digital storytelling [12], and these programs require digital literacy for multimedia editing and digital storytelling. Some studies required trained volunteers [26] or caregivers [23,27] to support people with dementia in developing stories. However, these settings are intended to enable people with MCI to adapt to existing environments and interactive tools rather than customizing the software to their specific needs (thus enhancing the usability of the storytelling application while simultaneously reducing learning costs). The applicability of these methods for memory retrieval among adults with MCI is currently not fully understood [28]. Therefore, our aim was to co-design a technology-based group memory retrieval intervention, which is a key form of digital storytelling intervention, to support social participation. We also investigated how to design user-friendly storytelling applications to manage digital resources and reduce learning costs for people with MCI.

### Theoretical Framework

Co-design pertains to the collaborative involvement of people (users and stakeholders) in the design of a product or service [29]. Co-design workshops for interventions for people with MCI involve people with MCI, caregivers, and therapists in designing the intervention programs, providing unique perspectives. Involving users in design sessions helps engineers and researchers better understand requirements. A co-design approach would be helpful in identifying the requirements for designing technology-based group memory retrieval interventions for people with MCI. In our study, the development of the storytelling prototype was guided by 2 behavioral and theoretical frameworks to raise participants’ awareness about their social health behaviors and tailor adequate actions for behavior change, namely the Behavior Change Wheel [30] and Theoretical Domains Framework (TDF). Beginning with the Behavior Change Wheel, a behavioral analysis enables intervention designers to select particular areas for exploration, thereby guiding the development of the digital storytelling approach. TDF deepens the understanding of psychological and behavioral factors influencing effective interventions. This integration aligned our application’s features with identified behavior change strategies, fostering a comprehensive approach to promoting social health. In addition, a communication strategy called MESSAGE (an acronym representing 7 key communication strategies, each encapsulated by its initial letter; within each strategy, specific suggestions are aimed at enhancing effective communication: maximizing attention [M], focusing on expression and body language [E], keeping it simple [S], providing support for their conversation [S], assisting with aids [A], getting their message [G], and encouraging and engaging in conversation [E]) [31] was used to support digital storytelling communication. Considering the critical importance of providing caregivers with effective tools to bolster memory and communication abilities in individuals with dementia [32], the implementation of specific communication strategies between people with MCI and the Huiyou application is paramount.

## Methods

### Ethical Considerations

The Swinburne University of Technology’s Human Research and Ethics Committee provided ethics approval for this research (20226525-11105) on September 26, 2022. All workshop participants provided written informed consent to participate and gave permission for their audio recordings and sketches to be used in publications. All methods were performed in accordance with relevant guidelines and regulations. We collected no identifying information from the research participants, such as their names or email addresses. Each participant was assigned a unique ID number to ensure their anonymity. In addition, we provided informed consent forms, including a project information sheet, to uphold their privacy rights.

### Research Team

The research team is a collaborative ensemble of specialists in human-computer interaction, user experience, and design, each contributing their distinct expertise to the success of the project. AAM has an extensive background in design research and human-computer interaction, and WL has engaged in and performed research on user experience extensively. DZ is a doctor of philosophy student specializing in design.

### Sample

A social work organization named Jingshilaonian, located in Beijing, China, expressed willingness to participate in this research. Situated within the Tiantongyuan community in Beijing, this institution specializes in providing psychosocial support, mental health services, and daily activity assistance to older adults, including those with MCI. Jingshilaonian played a vital role by assisting us with the recruitment and screening of people with MCI by administering the Montreal Cognitive Assessment, as developed by Nasreddine [33]. The inclusion criteria for people with MCI encompassed independent community dwelling, age >65 years, no visual or hearing impairments, and adequate reading ability. The exclusion criteria for people with MCI included significant neurological conditions, such as stroke or brain injury, because of potential confounding effects. Caregivers had no specific inclusion criteria. For therapists, the inclusion criteria were postgraduate qualifications and >3 years of experience in social interventions. To engage participants, we collaborated with the organization’s manager to distribute information sheets to people with MCI and their caregivers, inviting them to participate in the study. We co-designed a storytelling intervention with people with MCI (n=12), their caregivers (n=4), and therapists (n=5) in Beijing, China. The brainstorming stage and rough prototyping stage for the intervention were attended by people with MCI and caregivers in a group setting, and the user testing stage was attended by people with MCI and therapists. People with MCI (n=12) were aged 65 to 77 (mean 69.91, SD 4.20) years, and their average Montreal Cognitive Assessment-Chinese version score was 23.58 (SD 1.38). A total of 4 caregivers participated in the workshops. Table 1 summarizes the demographics of people with MCI.

**Table 1:** Demographics of participants with mild cognitive impairment (n=12).

Characteristic			Values
**Sex, n (%)**			
	Male	3 (25)	
	Female	9 (75)	
Age (y), mean (SD)			69.92 (4.20)
**Age (y), n (%)**			
	65-69	5 (42)	
	70-74	6 (50)	
	75-79	1 (8)	
**Educational background, n (%)**			
	Bachelor’s degree	2 (17)	
	High school	3 (25)	
	Junior high school	7 (58)	

### Procedure

#### Overview

**Figure 1:**
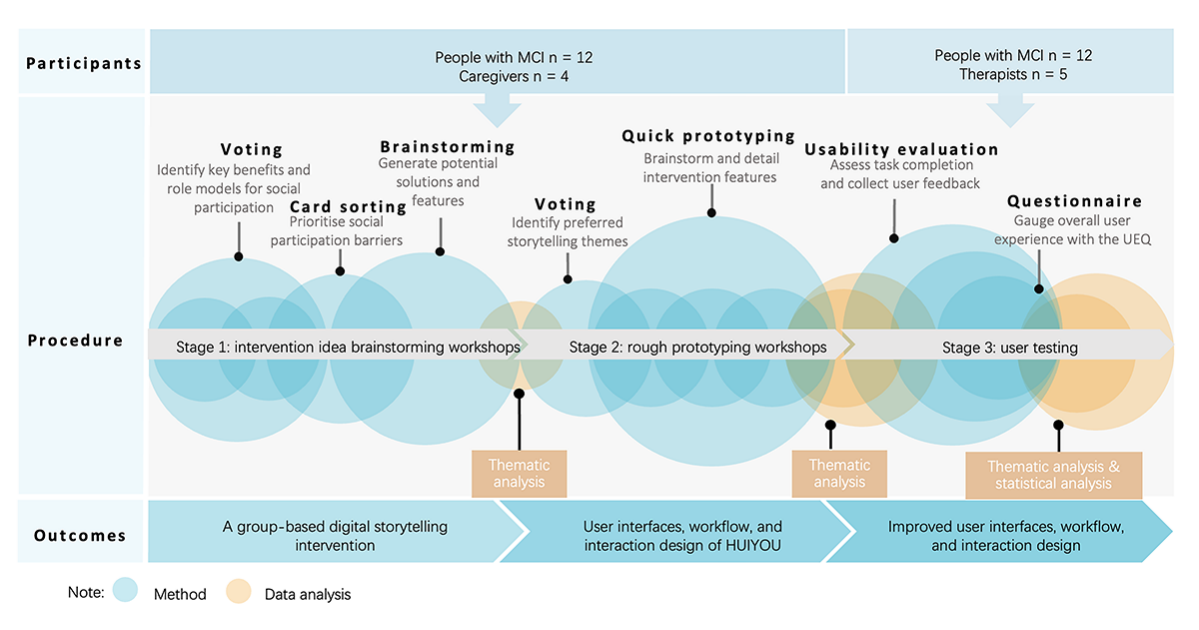
The process of data collection. MCI: mild cognitive impairment; UEQ: User Experience Questionnaire.

We adopted the co-design stages proposed by Robinson et al [34], namely scoping (stage 1), participatory design workshops (stage 1), and prototype development (stage 2). After developing the prototype, we conducted a usability evaluation (stage 3; Figure 1). The specific tools and procedures used can be found in the protocol paper [14].

#### Stage 1: Intervention Idea Brainstorming Workshops

In this initial phase, it took 45 minutes to uncover the essential aspects of significant social participation for people with MCI and their caregivers. This stage involved a sequence of 4 workshops designed to identify the most compelling benefits, role models, and barriers related to social participation. By prioritizing these aspects, we sought to deepen our understanding of the significance of social participation for people with MCI and enhance the quality of data for the subsequent phase. During each workshop, people with MCI and caregivers voted on the most compelling benefits and role models, and people with MCI selected 2 abilities that they wished to improve. These insights were critical for informing the design of our digital storytelling intervention.

#### Stage 2: Prototyping Workshops

**Figure 2:**
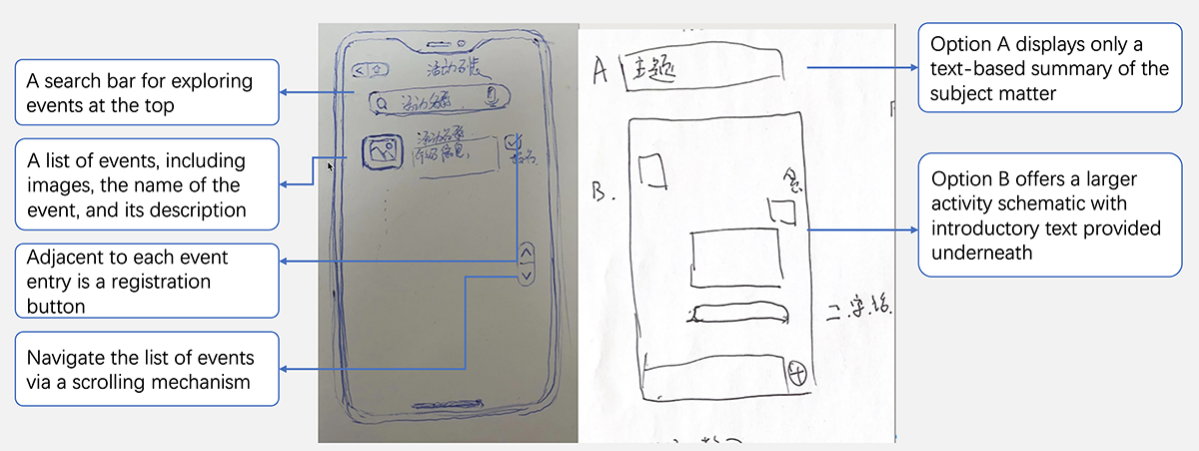
A sketch showing browsing and reminiscing topics and details.

**Figure 3:**
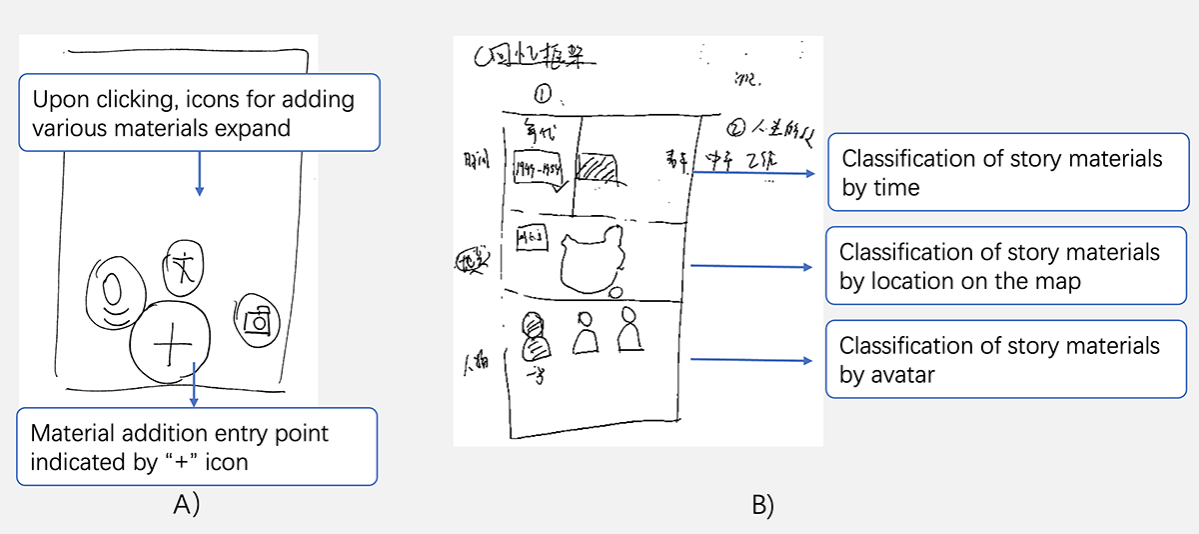
A sketch of (A) material addition entry point indicated by a “+” icon and (B) adding material description of story materials.

**Figure 4:**
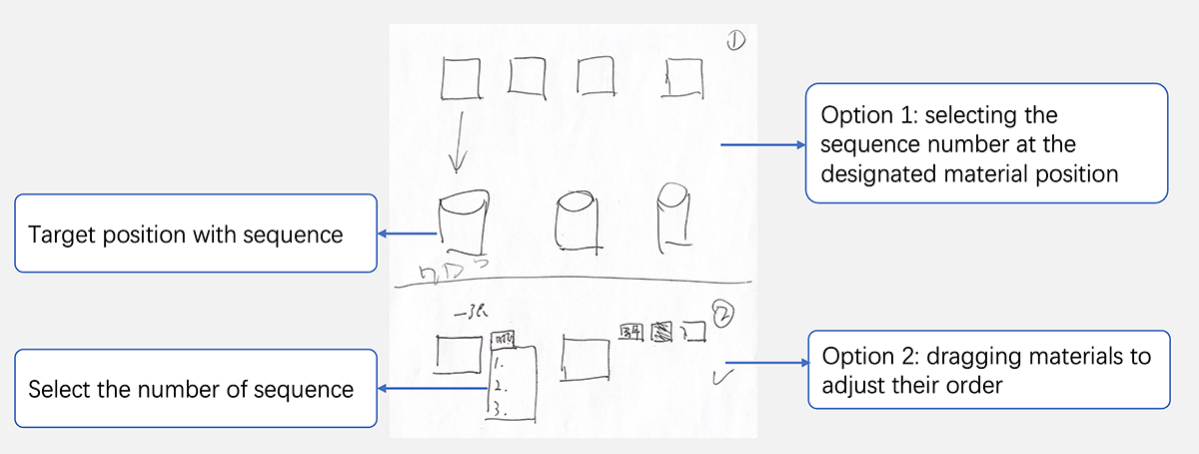
A sketch of changing the sequence of the content.

**Figure 5:**
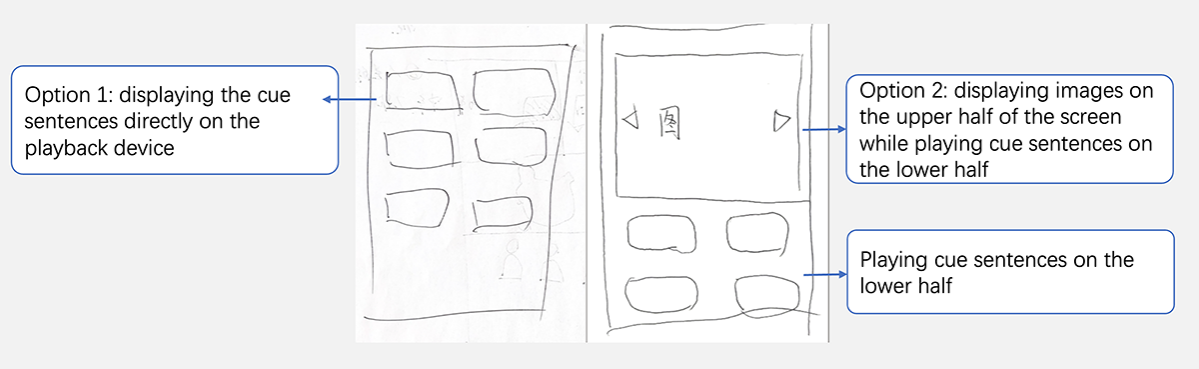
Cheat sheet example.

**Figure 6:**
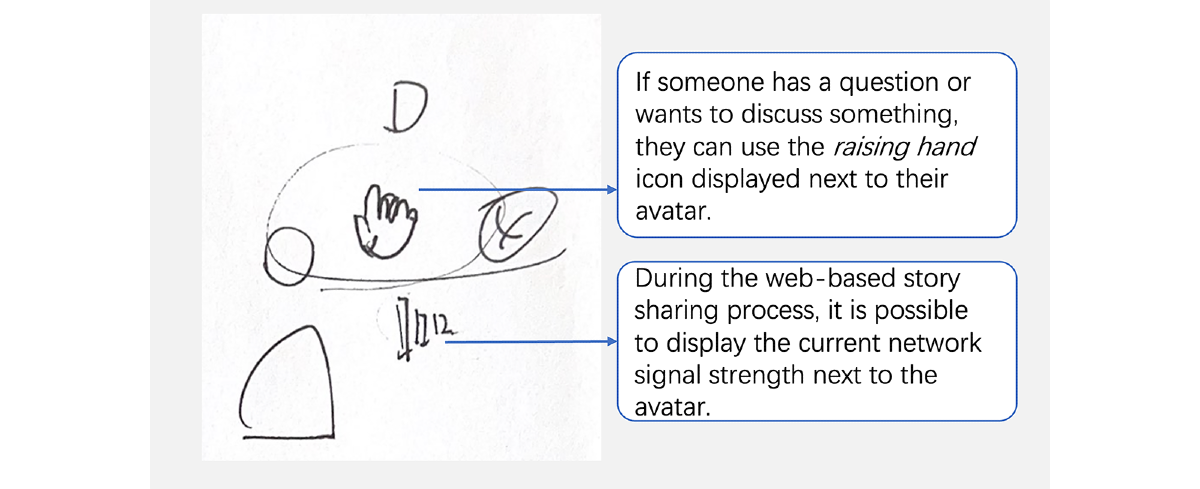
Participants can raise their hand with any question they wished to ask.

Building on the insights gained from stage 1, we focused on collaborative brainstorming sessions involving people with MCI and their caregivers in stage 2. The goal was to explore the design and interaction logic of the interface through 60-minute workshops. Digital storytelling interventions involve recalling past actions, events, and feelings using physical prompts. In addition to building on the insights gained from the literature review of digital storytelling interventions, we identified the main features of digital storytelling applications as story creation, memory retrieval, and story sharing [12]. Furthermore, we found that the themes of stories significantly impacted the storytelling experience [35]. Therefore, the research team proposed 4 primary properties for potential features of the digital storytelling intervention: identification of preferred storytelling themes, support for story material generation, memory retrieval, and story sharing. Participants were encouraged to describe, illustrate, or display sample interfaces on their mobile devices. Figures 2-6 were generated during the prototyping workshops by participants and facilitator DZ. The key objectives were to define the program’s main goals; propose relevant features, interactions, workflow, and interface elements; and create rough prototypes to visualize the intervention’s interfaces and interactions. The outcomes of this stage provided the foundation for designing the prototype of our digital storytelling application.

#### Stage 3: User Testing

We created the Huiyou prototype based on the insights and ideas gathered from stages 1 and 2. To evaluate the application’s usability, we identified 22 specific tasks aligned with its features and interaction logic. These tasks were meticulously selected to ensure that the intervention’s primary objectives, including enhancing social connection and community participation for people with MCI, could be effectively achieved. Participants, including people with MCI and therapists, engaged in usability testing sessions, where they completed these tasks and provided feedback on their experiences. The usability test session was 45 minutes long. Feedback gathered from these sessions included task success rates, concerns, and preferences. We also collected data on individual task completion rates and task completion with facilitator assistance. In our usability testing, we used a comprehensive approach to evaluate the features and subtasks of the Huiyou intervention. For instance, in testing the story sharing feature, participants were tasked with actions such as entering the speech mode, locating speech prompts, assessing speech length on the page, and identifying the screen projection button. If participants independently interacted with the interfaces successfully, the facilitator recorded it as task completion without assistance. However, if participants encountered difficulties in completing a task even after thoroughly examining the entire interface, the facilitator intervened by offering predefined tips, such as suggesting checking the bottom of the screen. In such cases where participants were able to successfully interact with the interfaces with the facilitator’s assistance, the facilitator recorded it as completion with facilitator assistance. Following this, we initiated an iterative design process to refine and enhance the user interfaces (UIs) based on participant feedback, ensuring the application’s usability and effectiveness.

### Data Collection

Qualitative data were gathered through interviews, sketches, and focus group discussions at different stages of the research via audio recordings. In stage 1, focus group discussions were conducted to analyze the prioritized aspects of social participation. Stage 2 involved brainstorming activities during which participants generated sketches and discussed the potential features of Huiyou. In stage 3, participants’ feedback was collected through self-reporting during usability testing and structured interviews. It is important to note that the Chinese version of the User Experience Questionnaire (UEQ) had previously undergone psychological validation [36]. A total of 22 usability tasks were used to evaluate task completion, providing a comprehensive approach to gaining valuable insights into the user experience of Huiyou and effectively assessing its usability.

### Data Analysis

Qualitative data analysis followed a thematic analysis approach [37,38]. Two individual researchers reviewed and coded the interview transcripts and organized the codes into themes using NVivo (version 12.0; Lumivero) [39]. Labels, such as “Stage 1-group 2-people with MCI number 2,” were used to distinguish different groups and participants.

In the design phase, the research team used the JiShiSheJi design to create the interface. After usability testing, a redesign phase was initiated, involving a detailed analysis of participant feedback and interview responses. These data were systematically summarized to identify improvement areas. An iterative design process was then used to enhance the UIs by incorporating participants’ feedback. This approach ensured that the final version of Huiyou aligned better with the needs and preferences of people with MCI.

Quantitative data analysis involved calculating completion rates using Excel (Microsoft Corp) to determine the average completion rate and feature rankings. The UEQ results were assessed using the UEQ Handbook [40], whereas the UEQ Data Analysis Tool Version 12 was used to compute mean values, SD, and CI for various usability dimensions of attractiveness, ease of use, efficiency, dependability, stimulation, and novelty. We also used Excel to calculate the mean values of task completion rates with and without assistance.

## Results

The key findings from each stage of this study are summarized as follows.

### Stage 1 Findings: Intervention Idea Brainstorming Workshops

#### Overview

Our exploration unfolded into 3 distinctive themes, each shedding light on a crucial aspect of participants’ experiences. We examined how social participation can be a powerful catalyst for enhancing physical health and fostering lifelong learning. Through the inspiring stories of confident older adults who wholeheartedly embraced challenges, our second theme showcased exemplars of unreserved social participation. Finally, we delved into the primary barriers people with MCI aimed to overcome, with a particular focus on memory, language expression, and the mastery of essential smartphone skills.

#### Enhancing Physical Health and Lifelong Learning Through Social Participation

Most participants with MCI (8/12, 67%) regarded social participation as an approach to maintaining physical health. They found that attending social activities can enhance their health status because they are less prone to catching colds when actively participating in social activities. People with MCI pay more attention to physical health; therefore, the benefits of physical health may attract people with MCI to participate in social activities.

Most participants with MCI (8/12, 67%) reported that engaging in activities with older adults positively impacted their physical well-being; these activities, such as exercise programs or outdoor group activities, helped improve their physical condition, increased their mobility, and supported their overall health. In addition, the social interaction involved in these activities afforded them a support system, along with accountability and the motivation to stay active and adopt a healthy lifestyle. In agreement with this, participants stated the following:

Engaging with others and staying socially active can help improve my physical health. It encourages me to stay active, spend time with people, and motivates me to maintain a healthy lifestyle.

Stage 1, G2-P3

Few participants with MCI (3/12, 25%) emphasized the value of lifelong learning and intellectual stimulation; participating in social activities provided these participants with a platform for communication, exploration, and the imparting of knowledge. By engaging in collaborative social activities and exchanging ideas with others, they were encouraged to continuously learn and discover new things. One of the participants commented the following:

Engaging in social activities can improve my mental health by allowing me to interact with others, receive emotional support, reduce feelings of loneliness, and enhance positive emotional experiences.

Stage 1, G1-P2

#### People With MCI Admire Peers Who Face Challenges Confidently

Most participants with MCI (9/12, 75%) admired older adults who displayed the confidence to attend various activities. Interacting with confident older adults allowed these participants to gain a new perspective on aging and life experiences; they were inspired to embrace challenges, overcome impediments, and approach life with a positive mindset:

They give me strength and inspiration, demonstrating passion and a positive attitude in caring for others, making me believe that I can still have a fulfilling and meaningful life even in challenging circumstances.

Stage 1, G3-P3

#### People With MCI Want to Improve Their Memory and Language Deficit and Smartphone Skills

Table 2 indicates that most people with MCI wish to improve their memory and expression of language, as participants mentioned that memory affects them frequently and that language expression can affect communication with their grandchildren. Of the 12 participants with MCI, 7 (58%) prioritized the improvement of memory, as this affected them the most:

I have poor memory; as I spoke, I forgot where I was talking. Therefore, I want to improve it first.

Stage 1, G2-P2

**Table 2:** The top 3 barriers identified (N=24).

Barriers to overcome	Votes^a^, n (%)
Memory ability	7 (29)
Language expression	5 (21)
Smartphone use	4 (17)

^a^Each person with mild cognitive impairment had 2 votes; in total, there were 24 votes from the 12 participants.

Of the 12 participants with MCI, 5 (42%) mentioned that their storytelling ability also affected communication, mainly when talking with their grandchildren:

My education level is limited, and I hope I can improve my language skills so that I can tell my granddaughter vivid stories.

Stage 1, G3-P1

Among the 12 participants with MCI, 4 (33%) felt that using a smartphone was difficult, and some (3/12, 25%) people with MCI believed that the requirement for the ability to navigate a smartphone might prevent them from further social participation:

As I get older, I cannot use many functions of my phone. I always have to consult others, but it is more convenient to learn them myself.

Stage 1, G2-P3

All the participants with MCI (12/12, 100%) selected smartphones as the suitable device on which to install the application because they all had access to a smartphone, with only a few participants reporting that they owned an iPad (Apple Inc); smartphone was viewed as the more favorable option, being more portable than tablets or computers:

I have a smartphone; if the application could be installed on my phone, it would be more convenient since I carry the phone all the time.

Stage 1, G2-P2

In summary, the benefits of social participation for people with MCI included the improvement of physical health through engagement in activities, the fostering of a support network, and increased confidence among older adults. Memory and language expression were the primary barriers they wished to improve and were crucial for their communication with their grandchildren. Furthermore, their smartphone skills required enhancement to facilitate continued social participation.

### Stage 2 Findings: Prototyping Workshops

#### Overview

Following the analysis of the findings from stage 1, the research team believed that the creation of a group-based digital storytelling intervention would be beneficial for improving memory, language expression, and smartphone use skills, and eventually, it could enhance social connection and participation. We have outlined the findings regarding the 4 primary features of the digital storytelling application as follows.

#### Preferred Storytelling Themes for Sharing With Others

All the participants with MCI (12/12, 100%) stated that they would like to share recent activities, including hobbies (as well as the changes they perceived around them), social activities, and current politics. Hobbies were most commonly cited because the participants were eager to share them with others and had invested much time and effort in them. The participants with MCI reported the following:

I enjoy content related to technology, as well as driving. I like observing different cars; whenever a related topic arises, I cannot stop talking about it.

Stage 2, G2-P2

Moreover, participants suggested receiving memory retrieval themes to ensure a well-defined topic to reminisce and allow them to gather relevant materials beforehand, expediting the process for smoother implementation.

#### Design of the Story Material Generation

After defining the storytelling themes, users could generate story materials by obtaining topics for memory retrieval, collecting materials, adding material descriptions, and integrating materials for further story sharing. The facilitator encouraged participants with MCI to create sketches; however, only 4 (33%) of the 12 participants with MCI were inclined to do so (Figures 5 and 6). Consequently, the facilitator assisted those who were unwilling to draw in visualizing their ideas.

Figure 2 presents a sketch for browsing a topic for memory retrieval; on the left side of the page, there are a search bar for exploring events at the top and a list of events, including images, the name of the event, and its description at the bottom. The user navigates the list of events via a scrolling mechanism. Adjacent to each event entry is a registration button. The page on the right offers 2 options for displaying the activity. Option A displays only a text-based summary of the subject matter. By contrast, option B displayed a more significant activity schematic with introductory text provided underneath:

For example, for my son’s wedding, just get a few photos, just two or three.

Stage 2, G2-P3

The material collection feature enhances content by providing explanatory details and enriches the collection by sourcing pictures from various internet platforms. Recording snippets of information further aids by capturing valuable insights. In addition to collecting the target content, participants also suggested recording fragmented information informally. Figure 3 presents a sketch of an entry point for the supplementary material at the bottom of the page, indicated by a plus icon, which reveals 3 input methods when clicked. The input methods include voice input, text input, and video input:

The function of voice recognition is very convenient. With just a press of a button, recording starts, and in the end, it can be converted into text for preservation, making it easy to view later.

Stage 2, G1-P2

The adding material description feature aims to optimize the collection of data by associating individuals with each story (Figure 3); for instance, the developmental stages of the country, timeline, and the type of activities connecting people with MCI with the country’s developmental stages and arranging them chronologically and by different activities for easy navigation. Participants recommended the provision of a framework of memories to aid them in refining the story; for example, participants could select the time stage, such as the founding of the People’s Republic of China in 1949, and the stage of their life at the selected time, such as youth, middle-age, working, or retired. The choice of location is provided using a map. Finally, participants can apply labels for characters, including their avatars and names. One of the participants explained this as follows:

We can label the key characters that appear inside.

Stage 2, G3-P2

I like to categorize these [materials] by age and objectively exist since I am willing to use classification methods that are specific, not easily confused, preferably objectively present, and not prone to misunderstanding.

Stage 2, G3-P3

The integrating material feature aims to create a cohesive memory retrieval experience by uploading selected materials to the topic in question and adjusting sequences to promote a seamless and engaging narrative flow (Figure 4). The participants provided 2 options: option 1 displayed the candidate photos in the first row, and below, each theme was listed, with the corresponding material dragged and dropped into the theme; and option 2 allowed users to select the order of presentation after choosing the related theme. One of the participants with MCI commented the following:

After uploading these photos, we need to associate them with the story to be told based on different story themes. Before sharing, we could easily change the sequence of the materials.

Stage 2, G2-P1

#### Memory Retrieval Activity Design Through Facilitation Strategies

The digital storytelling application uses various strategies to enhance the experience in the memory retrieval function. It has 3 features: a story presentation, group discussion, and story summary.

During story presentation, the digital storytelling application has cheat sheets for recall, minimizing interruptions by using timers, promoting interactive discussions, and creating permanent records. Mobile technology facilitates the easy capture of memories, forming meaningful connections with cherished life events. Preparing a cheat sheet with helpful tips can provide valuable guidance to ensure a smooth and engaging memory retrieval experience (as shown in Figure 5). The cheat sheet might present either keyword prompts or corresponding content. A cue word switches to the content of the corresponding material, depending on the selected content format:

Sometimes [my] memory is poor, and this software can provide a cheat sheet to prevent me from forgetting what I need to share at the moment.

Stage 2, G1-P2

During the session, avoiding interruptions allowed participants to immerse themselves fully in their nostalgic journey, and a visual timer helped them manage their time effectively:

You can only limit the time. One is that most of the time, how many people cannot all stand on the same question, or you cannot all stand on your own time for a few minutes.

Stage 2, G4-P2

During a group discussion, the approach integrates memory retrieval with open discussion groups. Encouraging participants to pose questions by raising their hands fosters an interactive and dynamic environment (Figure 6). During the presentation, each participant has an avatar, and the Wi-Fi signal strength is displayed next to the avatar. If the participant has a question they wish to ask, they may raise their hand, and a small, raised hand icon is displayed next to their profile. These discussions can be informal, fostering a sense of engagement in shared experiences and collective memories:

Ask each other questions. This form is quite good. Ask each other because it is to discuss different opinions on a subject, and this is the best way. Yes, if there are no different opinions, this question may be a little biased.

Stage 2, G3-P3

I want to ask questions. If you want to ask questions that I am interested in, I can also raise my hand and answer them.

Stage 2, G1-C1

The story summary feature aims to make memory retrieval more tangible; facilitators can generate records or memories from the discussions, providing a lasting and meaningful resource for participants to cherish and revisit:

Currently, mobile phones are very convenient. When I take a group photo and see everyone's photos, I can recall what happened at that time. I also like to keep a diary and can simply remember one or two sentences.

Stage 2, G4-C1

#### Story Sharing to a Broader Audience

To share the records of their recollections with the desired person or group, participants suggested using WeChat (Tencent Holdings Limited), which allows for the convenient and efficient sharing of memories, ensuring that the memory retrieval experience can be cherished and enjoyed by those involved. Most participants with MCI (8/12, 67%) wished to share this experience with certain persons or groups, primarily through WeChat:

Based on different themes, [we can] send today’s activities to corresponding people, such as those with common interests or children. Share with them through WeChat and let them know the latest situation as well.

Stage 2, G2-P3

However, one of the people with MCI showed no interest in sharing the content with others:

At this age, I’m not willing to share with more people; at least subjectively, I have no intention to please anyone.

Stage 2, G1-P2

In summary, Table 3 outlines the key functionalities and features of the memory log system, along with valuable suggestions for their implementation. To generate materials, the inclusion of memory topics aids in focused preparation. Collecting materials might include adding explanations to materials, collecting pictures from the internet, and the informal recording of fragments of discussions. While adding material description, users could add details such as time, location, and key characters to the material. Users could integrate materials into each presentation by changing the sequence of materials. During memory retrieval, facilitators can benefit from cheat sheets, minimize interruptions, and use visual timers in presentations. Open discussions, hand raising, and interactive dialogues further enrich the experience. Summarizing these records enhanced memory retention. Sharing options, such as WeChat, extend the impact beyond the scope of the audience.

**Table 3:** The main features of Huiyou identified from stage 2 (ie, prototyping workshops).

Functionalities and features		Suggestions to implement
**Generating story materials**		
	Obtain a topic for memory retrieval	Inform the topic and collect relevant materials in advance
	Collect materials	Add a description to each record Collect pictures from others via the internet Casually record fragmented information
	Add material description	Add essential character to each material Link to the sequential steps of the country’s development Follow the timeline to show the content Categorize by different activities
	Integrate materials	Upload selected materials to the target topic Change the sequence of the materials
**Memory retrieval activity**		
	Story presentation	Prepare a cheat sheet to obtain tips Avoid interruptions during the presentation Set visual timer
	Group discussion	Combine memories and discuss Raise hands to ask questions Open discussion
	Story summary	Generate a record or memory
**Sharing story**		
	Share storytelling records with certain a person or group	Share the record via WeChat (Tencent Holdings Limited)

#### Developing the First Prototype of Huiyou

**Figure 7:**
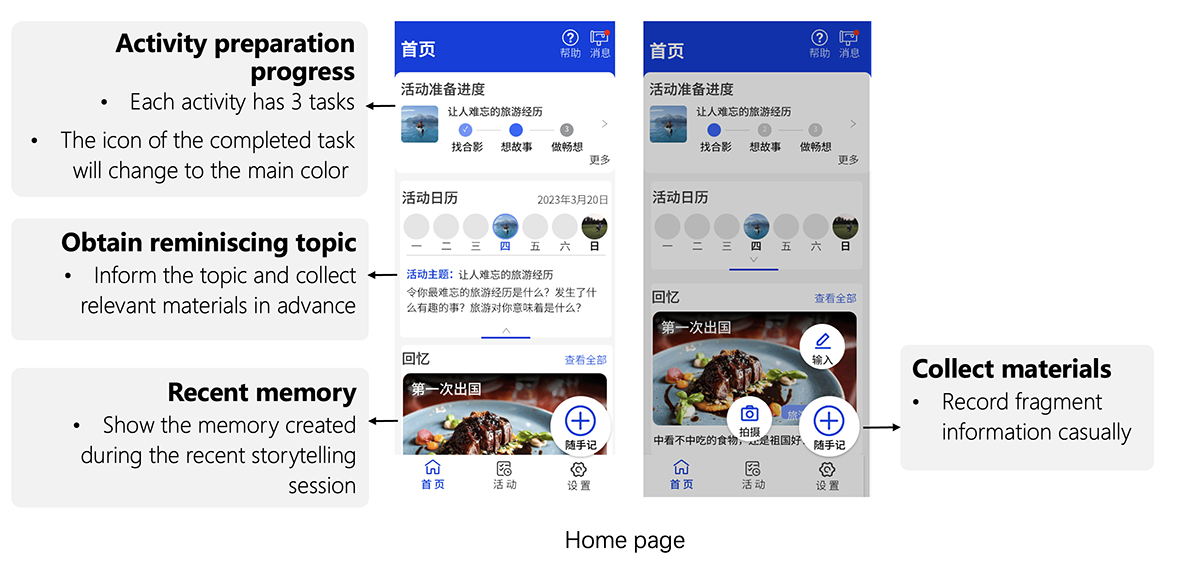
Huiyou prototype: home page.

The first digital prototype of Huiyou (Figure 7) was produced in JiShiSheJi [41], a web-based free software prototype design and development tool, following an iterative process of co-design workshops and feedback collected from the meetings. The software name 会友, Huiyou, derived from the Pinyin pronunciation, means “meeting new friends” in Chinese. It is inspired by a classic quote from Confucius in the Analects: “A gentleman seeks friendship through literature and reinforces goodness through friendship.” This statement emphasizes the idea that individuals cultivate friendships through literary exchange and support virtue through companionship. The name reflects a positive vision of fostering social connections through literature, friendship, and benevolence. Users can leverage technological means through the software to expand their social circles, facilitating deeper communication and connections with others. The name embodies the social nature of the software and its goal of promoting friendship. Huiyou effectively stores memories from daily life and encourages people with MCI to reminisce and discuss favorable memories with new friends. Huiyou has 2 main features: supporting people with MCI to conduct self-reflection daily (preparing materials with cues) on certain topics and facilitating group memory retrieval (presenting a story and promoting a discussion with group members). Two innovative aspects of Huiyou are embedded memory retrieval for capturing daily life and its ability to collect recent, valuable memories. Another such feature is combined self-reflection and group reflection to enhance social interaction during the discussion, in addition to sharing and participating in social activities outside the home.

**Figure 8:**
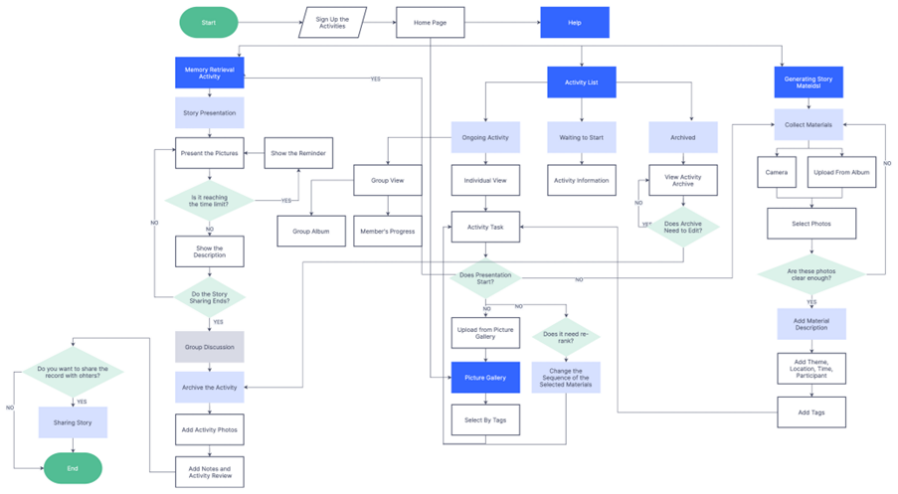
Workflow of Huiyou. A higher resolution version of this figure can be found in Multimedia Appendix 1.

As Figure 8 illustrates, the process of registering an activity on the Huiyou application begins with the user navigating from the home page to the activity page, where they can browse and select the activity they wish to register for; they then upload the material and edit it on the active page. When an event is about to commence, the user enters the event details page from the home screen. Next, they enter the activity and encounter a start screen for analyzing memories. The activity may be recorded and shared with others. If the user has any issues with the software, they can click the help button to discover operation guidelines.

### Stage 3 Findings: User Testing

#### Results From Task Analysis

Through this comprehensive usability testing stage, we identified minor areas of improvements in the interaction logic and interface layout to optimize user experience. In the 22 tasks, the participants scored an average success rate of 59% without assistance and 87% with assistance from the facilitator. Most challenges were identified to be associated with the direction of interaction; after knowing the direction, the success rate increased. Table 4 lists 5 tasks within the Huiyou digital storytelling application that demonstrated notably high usability, with completion rates exceeding 90% when performed individually. These tasks included “upload pictures” and “recording new materials,” both of which achieved perfect task completion rates of 100% with facilitator assistance. In addition, tasks such as “entering speech mode,” “find speech prompts,” and “insert a group photo after the event” also exhibited exceptional usability, with 92% individual task completion rates and 100% task completion rates with facilitator assistance.

This effectively communicates that within the Huiyou digital storytelling application, 5 tasks have a completion rate ≤75%, indicating the need for improvement. Tasks such as “add material description,” “browse registration activities,” and “switch the view of the activities” exhibited relatively lower individual task completion rates of 8%, whereas the presence of a facilitator notably enhanced completion rates to 58%. Similarly, the “judge whether the speech is too long on the page” and “enter the activity interface” tasks had initial completion rates of 25%, which significantly improved to 75% with facilitator assistance. These findings emphasize areas where the application’s UI and task guidance may benefit from refinement to ensure a more user-friendly and accessible experience, particularly for people with MCI.

**Table 4:** Task descriptions and completion rates of people with mild cognitive impairment (n=12).

Features and task			Task completion without assistance, n (%)		Task completion with facilitator assistance, n (%)
**Generating story materials**					
	Upload pictures	11 (92)		12 (100)	
	New materials for text input	6 (50)		10 (83)	
	New materials for voice input	7 (58)		10 (83)	
	Record new materials	12 (100)		12 (100)	
	Add material description	1 (8)		7 (58)	
	Add material tags	9 (75)		11 (92)	
	Edit material permissions	5 (42)		11 (92)	
	Enter my material	9 (75)		11 (92)	
	Change the sequence of materials	7 (58)		12 (100)	
	Browse registration activities	3 (25)		9 (75)	
	Enter the activity interface	6 (50)		9 (75)	
	Switch the view of the activities	1 (8)		7 (58)	
	Sign up for activities and add groups	5 (42)		9 (75)	
	View task progress	6 (50)		12 (100)	
	Enter task	10 (83)		11 (92)	
**Memory retrieval activity**					
	Enter speech mode	11 (92)		12 (100)	
	Find speech prompts	12 (100)		12 (100)	
	Judge whether the speech is too long on the page	3 (25)		9 (75)	
	Find the screen projection button	9 (75)		12 (100)	
**Sharing story**					
	Insert a group photo after the event	11 (92)		12 (100)	
	Use help features	4 (33)		10 (83)	

#### Results From the UEQ

**Figure 9:**
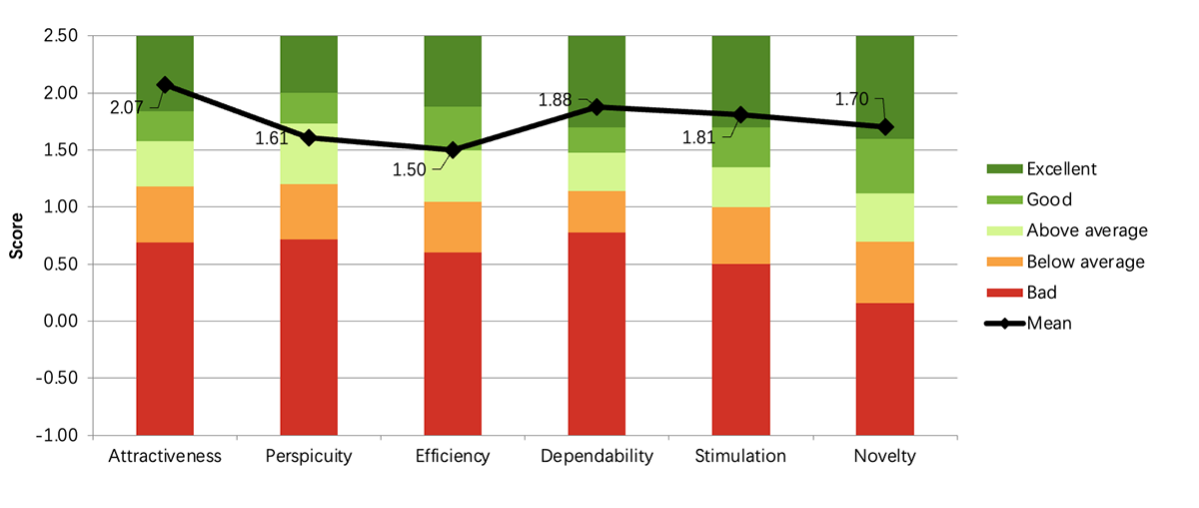
User Experience Questionnaire benchmark diagram for the Huiyou application.

When comparing the ratings to the UEQ benchmark [36], it was noted that the application falls within the average and above range, suggesting that there is room for improvement. The combined ratings for all users, including both people with MCI and therapists organized by the value of each UEQ item can be seen in Figure 9, wherein the average score pertaining to attractiveness is 2.073 (SD 0.82), ease of use is 1.609 (SD 1.15), efficiency is 1.5 (SD 0.85), dependability is 1.875 (SD 0.76), stimulation is 1.813 (SD 0.96), and novelty is 1.703 (SD 1.33).

In the results obtained from the UEQ (7-point positive and negative scale; Figure 9), participants with MCI expressed favorable opinions about Huiyou, perceiving it as an enjoyable (mean 2.7, SD 0.6), supportive (mean 2.3, SD 0.7), clear (mean 2.5, SD 0.7), and friendly (mean 2.3, SD 0.8) application. However, they found learning challenging (mean 1.5, SD 1.9) and somewhat complex (mean 0.9, SD 2.1).

#### Results From Usability Testing

On the basis of the interviews conducted and feedback collected during usability testing, we identified 15 usability issues to improve (Table 5). These issues were linked to the features of the application, including the home page and navigation, sign-up activities, adding material descriptions, changing the sequence of selected materials, sharing stories, uploading material, UI and design, and picture gallery. The main change was made to the home page (resolving the first usability issue in Table 5), addressing the user feedback that there was a lot of information on the home page and that the activity entrance was not clear, making it difficult to locate it quickly. One of the participants with MCI mentioned the following:

I’m not sure how to find the activities I want to participate in. The location of the activities should be more prominent. Those generated memories don’t necessarily have to be on the first page.

Stage 3, P3

Therefore, notable enhancements included moving activity records to a separate tab, thereby streamlining the navigation and organization processes. In addition, more activity entries were added to the home page, providing users with a broader range of options. We followed all recommendations to redesign the Huiyou application.

**Table 5:** Usability issues and recommendations.

Features and issues			Recommendations
**Home page and navigation**			
	There is a lot of information on the home page, and the activity entrance is not clear	Show the activities list. Each activity may have a different status: ongoing activity (in a dark green rounded rectangle) and waiting to start (in a gray rounded rectangle) Move the entrance of the “casually record fragmented information” feature from being suspended in the lower right corner to being fixed in the lower center	
	Category activity is hard to find	Move activity records to a separate tab Add more entries on the home page	
**Sign-up activities**			
	Activity registration setup issues	Display activity registration first, followed by recent activities	
**Adding material description**			
	Unable to understand the edit permissions	Change the heading to “invite others to edit”	
	Unable to find materials and view photos that have already been uploaded	Integrate the image library and add tags Allow easy switching and searching for images	
	Unable to understand content edit functions	Change to content description, integrating the description on 1 page, including time, location, characters, events, and others User should be able to add pictures’ tags from material, including family, friend, sightseeing, group photo, and item	
**Changing the sequence of selected materials**			
	Unable to sort materials	Provide operating instructions and make the font color of the instructions more eye-catching Provide support for 2 sorting methods: drag and click	
**Sharing story**			
	The “share the screen” button is too small	Make the screen projection button larger and highlight the color more prominently	
	Face-to-face discussions require no “raise hand” button	Remove the “raise hand” button for inquiries in face-to-face discussions	
**Uploading material**			
	No need to find internet resources	Delete the option of “upload material through internet”	
**User interface and design and picture gallery**			
	Need colorful and simple icons	Update icons to be colorful, eye-catching, and more solid	
	The return icon is not prominent	Increase the size of the return icon Change the dark background of the “help” icon to a lighter background	
	Keyboard input and voice input	Default voice input	
	The picture gallery contains too much information	Reduce the number of images displayed in galleries and preset some images in advance	

## Discussion

### Principal Findings

This study outlines the process and outcomes of co-designing and prototyping Huiyou, a digital storytelling application intended to facilitate social engagement and enhance the cognitive well-being of people with MCI in community-based settings. Insights collected from usability testing shed light on both strengths and areas for improvement in terms of user-friendliness and accessibility within the application’s design and functionality. Furthermore, some functions have been simplified, including the path for uploading material without internet resources. People with MCI may take 15 minutes to collect the materials they want to present and 10 minutes to present their stories. Each story is original, proposed by people with MCI, and they may ask for the support of volunteers or caregivers. The storytelling process has 2 phases: preparation of materials and memory retrieval. Emphasizing the recollection of recent memories was found to encourage social engagement and foster a sense of belonging. Huiyou, the storytelling application used in this study, facilitated material generation by providing preset content prompts and allowing independent material collection, setting it apart from other interventions. During the memory retrieval activity, participants used personalized cues and multimedia elements, triggering meaningful conversations and connections. This study’s findings suggested that storytelling themes for people with MCI should revolve around recent positive experiences and significant life periods. Notably, the application scored highly in attractiveness, dependability, stimulation, and novelty, although it required ease of use and efficiency enhancements. Recommendations for interface design included emphasizing crucial elements, minimizing cognitive complexity, and streamlining information presentation for improved user accessibility and experience.

### Comparison With Prior Work

This research highlights the importance of selecting storytelling themes that evoke recent positive experiences for people with MCI in China. The emphasis on significant life stages and changes, excluding marital experiences, is supported by existing literature [37]. Surprisingly, the study observed a keen interest among older male adults in political subjects, deviating from expectations based on previous research on political engagement among older Chinese adults [42]. It is proposed that prioritizing recent positive memories through Huiyou can encourage social engagement, fostering a sense of belonging and participation among people with MCI. Huiyou facilitates the storytelling process for people with MCI through self-collected materials rather than preset content prompts [43,44], engaging them to share their stories. According to the Capability, Opportunity, and Motivation–Behavior (COM-B) system, Huiyou aims to enhance psychological capability, create social opportunities, organize social activities regularly, and reinforce reflective and automatic motivations. In addition, the following TDF components were embedded in the prototype: skills, social role and identity, beliefs about capabilities, goals, memory, attention and decision processes, and social influences. For example, Huiyou incorporates behavior change strategies in the TDF that involve social influence [45], such as group discussions that monitor group progress. As the therapists said, these design features can foster peer pressure, enhancing adherence to task completion and effectively facilitating the establishment of social connections. During group discussions, Huiyou implements the MESSAGE communication strategy to involve people with MCI actively. For instance, it allows users to add notes to materials, which are then displayed on the screen during memory retrieval. Unlike other interventions, Huiyou enables users to collect and arrange materials themselves, enhancing their sense of accomplishment and reducing the need for external support. The application’s approach of facilitating material generation and arranging sequences aims to stimulate positive memories and encourage active engagement during the memory retrieval process. Therefore, Huiyou supports people with MCI in arranging the display sequences of materials, as OurStory does [23]. In memory retrieval activities, a substantial number of stimuli are prepared in advance and presented randomly to prompt older adults to narrate stories [46]. However, this approach relies on the divergent thinking abilities of older adults. Unfortunately, this method does not support the possibility of multiple stimuli coming together to form a more complete story. Unlike older adults with dementia, people with MCI possess the autonomy to select cues for their storytelling, fostering interpersonal connections and evoking positive emotions [47]. Personal topics serve as effective memory tests, enhancing storytelling and social memory [48]. Huiyou uses visual cues, music, and various technological platforms to stimulate memory retrieval and trigger discussions among older adults. By encouraging the recall of recent positive memories and promoting group discussions [49], Huiyou enhances the confidence of people with MCI, empowering them to actively participate in social interactions and community activities. Group discussions not only provide opportunities for self-expression but also foster reflection and inspiration from shared social experiences. Sharing recent memories via Huiyou fosters dialogue and active social engagement among individuals with MCI. They can either share their stories during group reminiscing sessions or record and distribute their memories to family and friends. Unlike some interventions that lack a structured approach to memory retrieval [50], Huiyou allows for the systematic recording and sharing of stories [51], primarily using WeChat as the chosen platform for its broad reach among friends and family.

### Usability Issues of Huiyou

On the basis of the UEQ benchmark results diagram, Huiyou earned a place in the “excellent” category [36]. These insights serve as invaluable pointers for refining the user experience of the application. People with MCI typically adhere to a top-to-bottom, left-to-right reading pattern [52], emphasizing the importance of placing essential interactive elements at the center or the top of the screen. Furthermore, adopting a more noticeable design approach, such as incorporating colored buttons, may be beneficial. The cognitive complexity experienced by people is influenced by the quantity of content displayed on a single screen. Even subtle variations in interface design demand additional information processing time for people with MCI. To mitigate cognitive strain, it is advisable to minimize the amount of information displayed on each screen and segment tasks into more manageable steps.

### Limitations

One limitation was the small number of participants with MCI throughout the study. To mitigate the impact of a small sample size, the research team invited the same participants to engage in multiple stages of the study, gathering diverse research data, including interview outcomes, hand-drawn interfaces, and usability task data, and introduced the perspective of caregivers of people with MCI. Reusing the participants was an efficient approach to prototype development and, to some extent, mitigated the impact on people with MCI as well as clinician time. Another limitation was that the Huiyou prototype was redesigned based on the participants’ feedback; however, in this study, we did not evaluate the refined prototype. In the future, field testing with people with MCI will be used to measure the effectiveness of the tool in improving social participation.

### Conclusions

We described the co-design processes of developing a digital storytelling intervention, Huiyou, in collaboration with people with MCI and caregivers. We then evaluated the user experience of the application based on the feedback of people with MCI, caregivers, and therapists. Huiyou incorporates story-sharing themes that align with the needs of people with MCI in China. These themes foster common topics and evoke positive emotions without delving into excessive privacy. Unlike traditional reminiscence therapy, it is confined to memory enhancement during intervention sessions, neglecting other potential intervention times. Huiyou transforms reminiscence into an everyday activity that individuals can engage in at their own convenience. This provides more opportunities to collect and cherish fond memories. Huiyou excelled in usability testing, earning an “excellent” rating in the UEQ benchmark for attractiveness, reliability, stimulation, and novelty. However, there is room for improvement in accessibility and efficiency. By combining social participation with the fostering of relationships and the stimulus to contact friends, the application not only promotes individual well-being but also meaningful social interactions and maintains vital relationships for people with MCI.

## Appendix

Multimedia Appendix 1 Higher resolution version of Figure 8.

## Abbreviations

COM-B: Capability, Opportunity, and Motivation–Behavior
MCI: mild cognitive impairment
MESSAGE: maximizing attention, focusing on expression and body language, keeping it simple, providing support for their conversation, assisting with aids, getting their message, and encouraging and engaging in conversation
TDF: Theoretical Domains Framework
UEQ: User Experience Questionnaire
UI: user interface
