# Supplementary figures and images for: Digital Storytelling Intervention for Enhancing the Social Participation of People With Mild Cognitive Impairment: Co-Design and Usability Study
Source: JMIR Aging. 2024 Jan 17;7:e54138. doi: 10.2196/54138 (PMC10831696; doi:10.2196/54138)

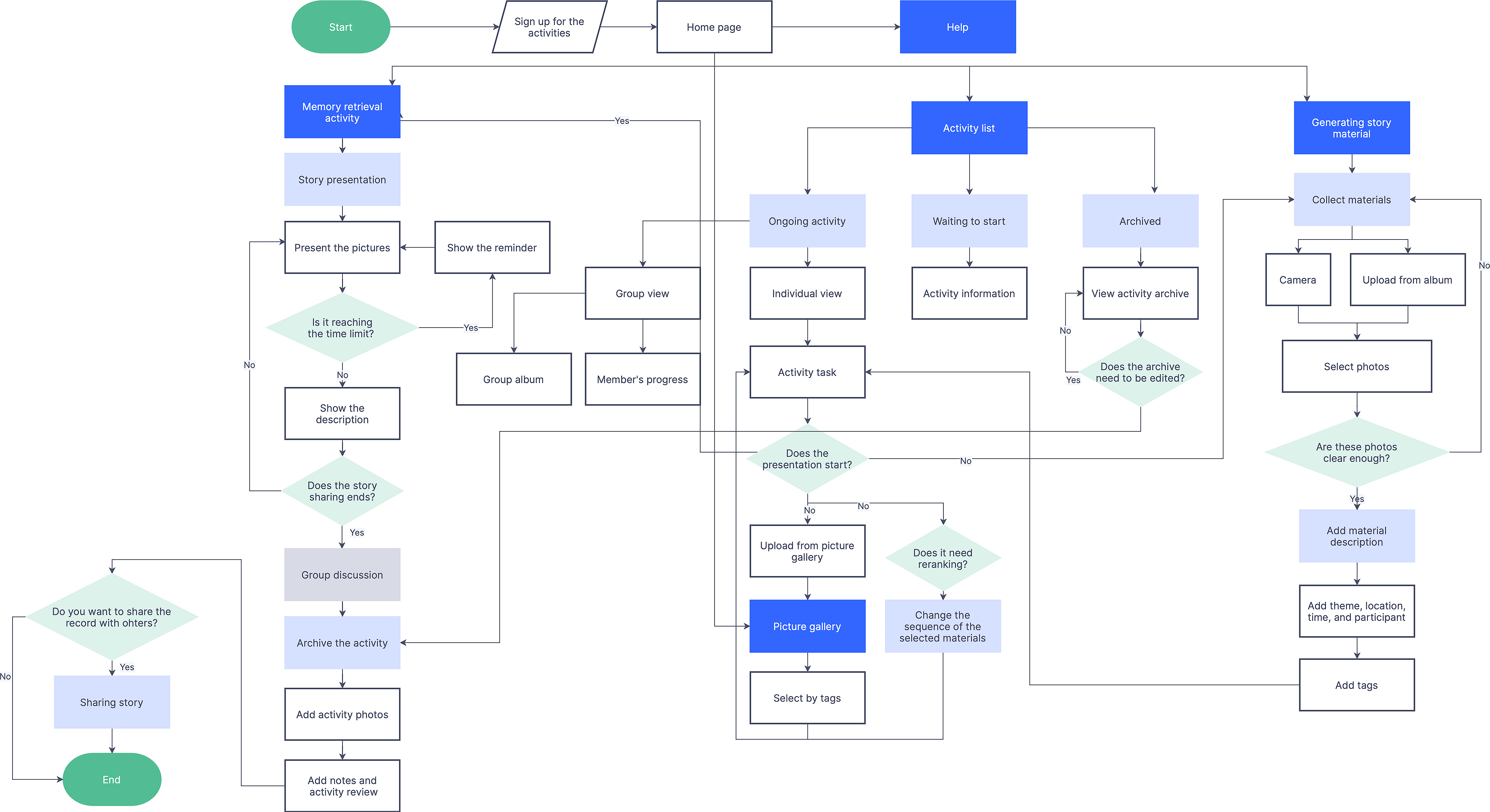

Supplement: Multimedia Appendix 1 [file aging_v7i1e54138_app1.png]
